# Towards a history of global hospital governance, late nineteenth century to mid-twentieth century: a contribution to a history yet to be written

**DOI:** 10.1590/S0104-59702024000100051

**Published:** 2024-10-14

**Authors:** Céline Paillette

**Affiliations:** i Scientific secretary, History Committee/French National Institute for Health and Biomedical Research; PhD student, Université Paris 1 Panthéon-Sorbonne. Paris – France; celine.paillette@inserm.fr

**Keywords:** Hospital, Governance, Global health, International organization, Innovation, Hospital, Governança, Saúde global, Organização internacional, Inovação

## Abstract

This article draws on the recent historiography in international relations and global health, as well as selected archives, to propose some milestones for a history of global hospital governance from the late nineteenth to the mid-twentieth century. How can we identify a system of hospital governance, including formal and informal arrangements, on a global or sub-global scale? Through the lens of the International Hospital Association, founded in 1931, we highlight the significance of internationalization processes, relationships with other international organizations, transnational interpersonal networks, and forces structuring innovation. This document provides a preliminary starting point for discussion and encourages further research.

## Resumo

O artigo baseia-se na historiografia recente das relações internacionais e da saúde global, bem como em arquivos selecionados, para propor alguns marcos para uma história da governança hospitalar global do final do século XIX a meados do século XX. Como podemos identificar um sistema de governança hospitalar, incluindo acordos formais e informais, em escala global ou subglobal? Por meio das lentes da International Hospital Association, fundada em 1931, destacamos a importância dos processos de internacionalização, das relações com outras organizações internacionais, das redes interpessoais transnacionais e das forças que estruturam a inovação. Esse documento fornece um ponto de partida preliminar para a discussão e incentiva pesquisas adicionais.

At the tenth conference of the European Society for the History of Sciences, held in Brussels in September 2022, Isabel Amaral and Monique Palma organized a session devoted to the history of the hospital from two perspectives: a contrast between the local and the global and a focus on medical and/or health “crises.” From biomedical to socio-professional dimensions, the approach was holistic and comparative, based on case studies from cities as diverse as Porto and Rio de Janeiro. The aim was to take the hospital as a historical object embodying the specificity of each institution, while questioning the common characteristics and evolutions – organizational objectives, material and human resources, architecture etc. – of a hospital “world” open to society (Risse, 1999). The cross-cutting issue of governance, as addressed during the session, referred mainly to the relationship between central authorities – administrative, ministerial, and other bodies involved in public health – and the hospital. Other topics discussed included how crises served to reveal the functions and dysfunctions of the hospital world, what local and central policies could be used to manage it, and, more importantly, the need to draw on past experience for present and future management of epidemics and health risks in the hospital environment. Further original perspectives on the political and material sides of this history were brought by a focus on heritage issues and the sense of appropriation of hospitals by all or part of their citizen body, caregivers, and patients. The session provoked the participants to consider hospitals in their wholeness, taking them out of the enclosed world in which certain histories have confined them (Granshow, 1993, p.1180). This special issue is the result of this Brussels meeting, and bears witness to the wealth of issues and historiographical developments underway in the vast field of hospital history. Coming from the history of diplomacy and international organizations on global health and epidemics, we have taken advantage of the richness of these debates to propose some possible guidelines for a history of the global hospital governance.

Positioning the hospital between global and local also means making a global history of hospitals. *From Western medicine to global medicine: the hospital beyond the West*, edited by Mark Harrison, Margaret Jones, and Helen Sweet (2009), explores the role of the modern hospital as a vehicle for Western medicine beyond the Western world, moving away from a monolithic, Eurocentric approach and focusing on local circulations. A total history of the hospital, as called for by Günter B. Risse in 1999, could be enriched by these global perspectives (Risse, 1987). Some researches have already opened up paths to understanding the history of hospitals, considering the importance of interpersonal relations and innovation processes and taking in international and transnational dimensions in the context of a metropolitan area, such as Mexico City (Agostoni, 2021) or a whole country, such as Argentina (Müller, 2018).

More specifically, we believe that the history of global hospital governance deserves to be documented. Studies in the history of medicine and health that have participated in the global historical turn since 2000-2010 (Harrison, 2013, 2015) have historicized the emergence of global health, understood as the transformations of health governance at the international scale (Packard, 2016; Gaudillière et al., 2020; Cueto, Rodogno, Bourbonnais, 2020). Put simply, governance encompasses the mechanisms that produce international regulations around a common interest, involving private and public stakeholders according to ongoing negotiation processes in protean formal and informal multilateral forums (Iriye, 2002). While the notion of governance is generally associated with the post-1945 period or even with the neoliberal turn of the 1980s, the processes that characterize it have been developing in the field of health since the mid-nineteenth century. A number of works highlight the role of international health organizations, notably the World Health Organization (WHO) and the League of Nations Health Organization (LNHO) (Cueto, Brown, Fee, 2019; Borowy, 2009; Weindling, 1995). And while the history of international organizations is only one aspect of what the concept of governance covers, these works reveal the makings of a complex multipolar international health system, associating public and private stakeholders (Tournès, 2022, chap.3), since the end of the nineteenth century.

Our aim is therefore to examine and cross-reference historiographical fields that, in our view, still do not intersect adequately, in particular the history of public health and the history of international relations. Based on a selection of existing works and archival surveys, the aim is to identify avenues for understanding the implementation of hospital governance on a global scale, which is not to say a world scale (Osterhammel, 2014). To some extent, the global scale is still a rather complicated subject, but it is much more restricted than the world scale. Other fields in the history of technology and science have adopted a similar approach, particularly in the history of telecommunications (Laborie, 2018).

Consequently, the question posed is a simple one: how can we identify a system of hospital governance, including formal and informal arrangements, that encompasses all of the globe or part of it? How does the hospital world participate in the processes of health globalization and, conversely, how is it shaped by them? Based on recent historiography and archival surveys, this article proposes a number of paths that could contribute to a history of global hospital governance from the late nineteenth to the mid-twentieth century. We focus on three areas of investigation: international organizations, interpersonal networks, and innovation. Research carried out on the governance of telecommunications, on international economic regulations, and on labor has shown the validity of these approaches.

Our hypothesis is that international organizations – embodying as they do both processes of institutionalization of hospital relations and techno-scientific innovation, through its inclusion in international economic circuits – are valid entry points for constructing this history, which is interested in the regulation of hospital relations.

## In search of an international hospital organization

One of the first areas to cover in a history of global hospital governance is that of international organizations with an interest in hospitals. The history of one such organization, the International Hospital Association, is gradually being documented. On June 13 and 14, 1929, the first International Hospital Congress was held at the Ritz Carlton Hotel, in Atlantic City, New Jersey, USA, on the initiative of the American Hospital Association. This organization had been preparing for the creation of an international association for several years by establishing dialogue with hospital representatives in Europe, such as Quellet, head of the Department of Hospitals of Assistance Publique, in Paris (An International Hospital…, 1927). This international congress combined medical-scientific exchanges with moments of sociability, as was often common practice at international meetings. From the beginning of the month, delegates were invited to visit hospitals on the northeastern coast of the United States and even as far away as Montreal; to take part in excursions (e.g., to Niagara Falls); to attend dinners; and so forth. The tours were organized under the auspices of various North American hospital authorities, such as the superintendents of New York City hospitals (Comprehensive Program…, 1929). The congress took place against the broader backdrop of a major change in the management and administration of North American hospitals. Hospital physicians, practitioners, and administrators from some thirty countries made comparative assessments of hospital development in the Americas and Europe. On both sides of the Atlantic, these developments were scrutinized: comparisons were made of hospital use in social and quantitative terms; the relative proportion of private and public facilities; hospital architecture; the education and training of medical, nursing, and administrative staff; medical equipment and care protocols; the place and consideration of the patient; and, of course, costs and management.

In 1931, this experience was institutionalized with the founding of the International Hospital Association at a congress in Vienna, which was attended by a larger number of participants. The organization presented itself as an association of hospital professionals. Its mission was to provide an international forum for professional exchange on issues common to the hospital community, to which end it organized international hospital congresses every two years thenceforth. As in 1929, scientific presentations and debates went hand in hand with social events. In 1931, the American Express Company organized the stay of the congress delegates, enabling them to explore not only the International Exhibition of Hospitals but also Vienna, its castles, museums, galleries, and the surrounding area, as well as attending a performance at the opera house and at the Spanish Riding School; a real sightseeing tour (Comité International…, n.d.).

The 1935 International Hospital Congress in Rome followed a similar pattern, bringing together some five hundred delegates at the Palazzo Corsini. Among the technical discussions were questions of international law, such as the status of civilian hospitals in wartime in light of the Hague Convention. The congress included a new international exhibition of hospitals and tourist excursions and visits worthy of a diplomatic delegation: in addition to the historical sites, there was a visit to the headquarters of the Fascist Party, a special audience with Pius XI, and a visit to a pavilion dedicated to new neurological therapies in the presence of the queen (Sand, 1935).

In addition to these events, the association organized training courses and published a quarterly journal, *Nosokomeion* (Donzé, 2021, p.105-106; Risse, 1999, p.472-475). This was the communication flagship of the international association and a unifying tool for the national associations. Articles published in several languages were accompanied by abstracts in English, French, and German. In addition to containing congress reports and the proceedings of special committees, the journal also provided information aimed at hospital professionals. The first volumes were devoted to the respective roles of physicians and nurses in hospitals, as well as economic issues and hospital architecture (Nosokomeion, 1932). A self-proclaimed “excellent instrument of study and propaganda,” *Nosokomeion* presented the progress of hospitals as a matter of “capital and international” importance, hospitals constituting “organs of social hygiene and the most important elements in safeguarding public health” (Nosokomeion, 1932, 1930). In this way, the International Association contributed to the achievement of a common goal, namely, the improvement of everyone’s health (Willis, Goad, Logan, 2018, p.3-11).

## The International Hospital Association in the network of health and medical organizations

While a monograph on the International Hospital Association has yet to be written, its history can nonetheless be traced in relation to other generations of international organizations and other internationalization processes involving hospitals. In this respect, the relational history of international organizations suggested by Pierre-Yves Saunier (2010) is still an essential reference. As for the period preceding the creation of the International Hospital Association, there are few traces of any kind of universal technical union – a term used to refer to the first generation of international organizations, which began to take shape in the 1860s (Herren, 2001; Iriye, 2002) – whose explicit object was the hospital. Alfred Fried’s *Annuaire de la vie internationale* (1906), which in the early 1900s listed private associations as well as international congresses and conferences, makes no mention of any international organization or association directly or globally concerned with hospitals. To find evidence of international concertation, it would be advisable to consider the various sessions of the International Congresses of Public Hygiene, which were held regularly as of the 1850s and occasionally addressed hospital issues (Rasmussen, 1990). Another path worth exploring is that of the international meetings and practices of trades and professionals involved in the hospital environment. In this respect, the concept of epistemic community could be fruitful. Clearly, the list of trades and professions will vary according to the professional category involved, beginning with nurses and doctors, but also considering administrative staff. It will also vary according to the specialization of hospital departments – surgery, neurology, psychiatry etc. – not to mention the fields of health engineering and architecture (Kisacky, 2017, p.344). The work of standardization in the field of surgery was a benchmark for the promoters of the internationalization of the hospital issue, with the First Conference on Hospital Standardization being held in Chicago in 1917 (MacEachern, 1942, p.1242).

Returning to the context of the International Hospital Association in the late 1920s and early 1930s, it is worth asking the extent to which the hospital issue fell within the realm of intergovernmental health organizations in the interwar period. At that time, there were two organizations with a global vocation: the Office International d’Hygiène Publique de Paris (OIHP), founded in 1907, and the LNHO, officially created in 1923. As Donzé points out, the remit of the Health Committee of the League of Nations (LON) did include hospitals. Beginning in 1928, at the request of a delegate from Czechoslovakia, who was asked to submit a report to the Office’s International Committee, the OIHP launched an international survey on the territorial distribution of hospitals and modern hospital design (Donzé, 2021, p.105). This survey was one of a series of studies carried out by the OIHP on various medical topics – goiter, cancer, tuberculosis, fever etc. – in line with its mandate to gather information so that its members could benefit from the experience of other countries in health matters. The OIHP collected and pooled data through forms submitted to the national health authorities of the state members, which then passed them on to local administrators and stakeholders (Paillette, 2012). At around the same time, it is possible to observe the emergence of hospital issues in other international and regional health cooperation forums. On the other side of the Atlantic, the 8th Pan-American Sanitary Conference, held in Lima, Peru, in 1927, called for a study of the hospital situation in the United States (MacEachern, 1942, p.1244). The concerns and issues raised in Paris and Lima were similar to those discussed at the First International Hospital Congress, held in Atlantic City in 1929 and organized by the American Hospital Association. It therefore seems essential to analyze in detail the role of the latter and the networks and circulations that put the hospital question on the international health agenda. For the period between the two world wars, we cannot overlook the health division of the US philanthropic organization the Rockefeller Foundation, which became the most powerful global organization in the field of health and played a structuring role in the public health policies of the various European states (Tournès, 2022; Doyle, 2018).

The International Hospital Association’s activities were suspended during the Second World War. In the Americas, a decision was made at the 1941 Atlantic City Convention to favor transcontinental cooperation, which resulted in the establishment of the Inter-American Hospital Association. This quickly became a forum for consultation and cooperation among national hospital associations from countries throughout the Americas and soon received requests for affiliation with professional associations, intergovernmental organizations, philanthropic foundations, and religious hospital associations (MacEachern, 1942, p.1244). At this point, it is worth assessing what was of shared interest from the point of view of state health policy in hospital matters.

## Interpersonal networks in hospital internationalism

An analysis of the interpersonal networks of this hospital internationalism will not only shed light on the construction of global governance but will also flesh out this history (Borowy, Hardy, 2008). Malcolm T. MacEachern (1942, p.1242), president of the International Hospital Association from 1935, identified Florence Nightingale, who had celebrated the spirit of cooperation conveyed by the World Columbian Exposition in Chicago in 1893, as an instigator of international cooperation among hospitals. The speech by the iconic figure of nursing, at the end of a text written for the Conference of Hospitals and Dispensaries, is ultimately representative of the humanitarian impulses accompanying the internationalism of the late nineteenth century (Nightingale, 1894). But MacEachern’s point of departure is interesting insofar as it informs the construction of a hagiographic narrative of hospital cooperation by its stakeholders. Another trajectory to follow is that of René Sand, a pioneer of social medicine and a leading figure in public health in Belgium, who was involved in several international organizations. His involvement in the International Hospital Association may well shed light on the history of global hospital governance. In 1929, the prestigious journal *The Modern Hospital* illustrated the first International Hospital Congress with a photograph of the acknowledged fathers of the International Association – Goldwater, Cornley, and Corwin – along with René Sand on the Atlantic City boardwalk. As early as the 1920s, René Sand (1929) had liaised between the American Hospital Association and the Belgian Hospital Association, which was founded on the North American model. In 1927, at the League of Red Cross Societies in Paris, he chaired a meeting initiated by the American Hospital Association that brought together representatives from 11 countries, going on to be appointed chairman of the International Hospital Committee (Donzé, 2021, p.105; An International Hospital…, 1927). In 1931, at the founding congress in Vienna, René Sand approached Andrija Stampar, a member of the LON Hygiene Committee and another well-known figure in social medicine, to recommend the new International Hospital Association to Ludwik Rajchman, medical director of the Hygiene Section of LON (Stampar, 23 July 1931). As with Rajchman (Balinska, 1998), René Sand’s energy in addressing all forms of health internationalization is remarkable. He served as an intermediary and relay between several organizations: not only was he secretary general and technical advisor of the League of Red Cross Societies, he was also Belgium’s representative on the LON Hygiene Committee from 1934 and a member of the Technical Preparatory Committee for the World Health Assembly in 1947 (Borowy, 2009, p.471; Zylberman, 2004, p.7).

It is instructive to identify the individuals and trace the professional trajectories of those who operated at the intersection of different organizations. As mentioned above, the International Hospital Association gave a prominent place to moments of conviviality that might provide opportunities for informal negotiation. Investigations of family ties and close friendships also reveal the cosmopolitanism and endogamy of these circles, as epitomized by the “international” marriage of Alter, editor of *Nosokomeion*, to Christiane Reimann, secretary of the International Council of Nurses and editor of its journal (An International Marriage, 1934).

## The global structuring of hospitals through technical-scientific and economic networks

Several studies show how the circulation of sanitary and scientific knowledge (hygiene, bacteriology etc.), products and practices (anesthesia, antisepsis, nursing, etc.), and the introduction of technical equipment (radiology etc.) helped to reshape not only hospital care systems and architecture but also hospital management (Risse, 1999; Blume, 1992). In order to identify the global governance of hospitals, it is essential to identify the technical-scientific and economic networks that contribute to this circulation, as well as the strategies and means used to deploy them.

The health care industry is inextricably linked to the functioning, development, and mission of hospitals. In line with this, some recent work initiated in business history invites us to consider the role of the medical industry in the transformations of the hospital world (Donzé, Fernández Pérez, 2019; Perkins, 2017). In order to respond to political and social demands, the growing need for more equipment, personnel, and funding, and the multiplicity of other actors involved, including insurance companies, the hospital world borrowed management practices from the private sector. Malcolm T. MacEachern’s *Hospital organization and management*, published in 1935, became an influential reference work; reprinted several times, it helped standardize the organization of Western hospitals. According to MacEachern (1957, p.25), more than any other enterprise, the complex organization of the hospital must be based on economics. Working under the authority of a governing board, the administrator plays a critical role in managing a hospital’s human, physical, and financial resources to provide efficient and cost-effective care to the sick and wounded while ensuring their comfort and safety. Administrators are also responsible for opening hospitals to new medical technology and introducing the most appropriate innovations to achieve their goals (McEachern, 1957, chap.4). In this regard, the history of the organization of American hospitals can be traced back through the twentieth century (Stevens, 1989). In her analysis of the worldwide circulation of management practices in modern hospitals from the late 1800s to the 1930s, Paloma Fernández Pérez (2021) demonstrates the weight of the North American model in the Western world. Malcolm T. McEachern’s tenure as vice-president (1935-1938) and then president (1938-1945) of the International Hospital Association is an invitation to assess how this international organization influenced the development of these practices as of the 1930s.

The history of hospitals can be investigated in other ways. Pierre-Yves Donzé, for example, analyzes the development of the German multinational Siemens in the international medical equipment market, particularly the market for X-ray equipment, as well as its involvement in hospital building on a global scale in the 1930s. The historian shows how the company participated in the international promotion of what he calls the “functional” hospital, characterized by a double division of labor, verticalization (specialized departments on each floor), and horizontal arrangement (rooms, offices, technical services on each floor). Siemens contributed not only to the standardization of this hospital model, in collaboration with the International Hospital Association and LNHO, but also to the actual construction of the facilities (Donzé 2014, 2015, 2021).

The importance of hospitals, hospital economics, and their links with the medical industry was well understood by the promoters of international health organizations. Of particular interest is the project for a European Health Community proposed by the French minister of Health, Paul Ribeyre, in 1952 (Paillette, 2021). In contrast with the WHO Regional Office for Europe, the European Health Community project was for a supranational European authority and the creation of common markets in the field of public health. This project encompassed several dimensions that impinged directly on hospitals in Europe. One of these was solidarity between states, the idea being to offer patients the best care possible on a European scale by taking advantage of whatever hospital specialties each member state was most distinguished in. Another aspect was the economic conversion of hospital facilities such as sanatoria, whose use was declining with the development of other forms of tuberculosis treatment (Godde, forthcoming), to other purposes, including tourism. Finally, with regard to the material needs of hospitals, several common markets were envisaged, such as the practice of pooling hospital building materials and the creation of a common market for textile products (e.g., sheets and bandages) and certain technical and biomedical equipment. In this respect, French stakeholders were particularly concerned about competition from German (Siemens) and Dutch (Philips) companies, which dominated the electro-radioelectric sector (Note…, 10 Oct. 1952).

## Final considerations

### The very late emergence of global hospital governance or sectorization first?

There is every reason to understand the global governance of hospitals in the context of the globalization processes that began in the 1860s. The creation of the International Association of Hospitals in 1931, although a pioneering achievement, seems to have come late compared to the internationalization of medical and health issues – exhibitions, conferences, international congresses – and professional associations, and even more so if compared to the circulation of hospital models linked to missionary movements (e.g., Renshaw, 2005). Two dynamics structured this first period of globalization in the late 1800s and early 1900s. On the one hand, it seems that hospital issues that overlapped with the interests of medical specialties or professional categories were dealt with in the multilateral forums devised within these fields. As a result, the international governance of hospitals emerged by sector. How, then, did a sense of belonging to the hospital world become a common denominator at the international level, beyond reference to a particular trade or medical specialty? Parallel to this, there was a convergence of hospital standardization processes – care, products, equipment – and the development of a “medical business system,” which combined industrial sector expertise with medical care, which was able to help bring about an *esprit de corps* and a common interest (Perkins, 2017). However, the needs generated in terms of hospital management and administration could also generate competition between hospital medical professionals and their administrators. Different analyses of this could be usefully developed in the long term. In this respect, the advent of computerization and telecommunications in the 1960s provides ample opportunity for investigation of its impact not only medical technologies but also on hospital administrative management tools and staff practices (Ceruzzi, 1998).

### Going beyond North American global hospital governance

For the interwar period, this initial research allows us to put forward the hypothesis of North American global hospital governance, spearheaded by the American Hospital Association. A comparison with the role of the Rockefeller Foundation in the global field of health is needed, and the goals – beyond the common cause – of international hospital cooperation, extending as it did throughout the Americas and Western Europe, needs to be documented. In the hospital sector, the American Hospital Association could be the vector of “soft power” or US diplomacy of influence of the kind that has been analyzed in some depth in the global health sector (Tournès, 2022). We need to examine how this US influence also responded to requests for cooperation, and how it was reappropriated and resisted in different parts of the world. This influence still marks the landscape and is the subject of a conservation policy. For example, the hospital built in Saint-Lô, Normandy, after the Second World War, symbolized Franco-American friendship and the affirmation of the American hospital and medical model in France. The France-United States Memorial Hospital, which attracted 15 thousand visitors at its inauguration in 1956 and is still in operation today, is listed as a *monument historique* (Beisson, 2004).

### The hospital as a topic of interest for state diplomacy

Future work is needed to assess the role of governments in these hospital internationalization processes, although they tend to be more visible in intergovernmental organizations, through national delegations, than in hospital associations or industrial groups. However, state diplomacy does take an interest in hospital issues. In France, as early as 1909, the Quai d’Orsay had a department for French schools and works abroad, which took under its wing hospital issues that had previously been the responsibility of political departments (Baillou, 1984, p.73-75). In 2009, the Direction Générale de la Mondialisation (Department of Globalization) of the French Foreign Office took under its wing the issue of global public goods, including global health issues (Nicoullaud, Grasset, Masset, 2018). Similarly, a series of studies led by Iris Borowy (2016, 2017) shows the German Democratic Republic foreign policy’s involvement in hospitals during the Cold War, examples being the Carlos Marx Hospital in Nicaragua and the Metema Hospital in Ethiopia, where politics and humanitarian support intersected. Conversely, through partnerships, hospitals could lead and develop international strategies aimed at building a positive image, promoting excellence, and conquering new markets with “international patients.” They could also act as foreign showcases for national governments, following the example of the proliferation of “US hospitals” around the world under a wide variety of statutes (as early as 1899 in Lima, 1906 in Paris, 1915 in Shanghai, 1920 in Istanbul...).

Contemporary global crises also reveal the new stakeholders involved in shaping global hospital governance (Hanley, Meyer, 2021). Today, the role of patients and patient associations must be linked to the history of global hospital governance. Their impact on a global scale has been examined in the context of the AIDS epidemic (Chan, 2015), but there is still a need for historical work documenting their role in other contexts. Of particular interest is the role and advocacy of patient associations with health authorities, as well as patients’ place in the medical and hospital ecosystem and the business of care, in the manner of citizen consumers. The growing recognition of patients as full partners and stakeholders in hospitals and biomedical research could create a favorable context for the history of their place on a global scale. As a consequence, all those who do not have access to health care cannot be limited to a “health mission” approach. As another example, since 2022, the WHO Europe Office, concerned about the risks associated with covid-19 and then the war in Ukraine, has put the hospital on its agenda. The general crisis of hospitals in Europe, highlighted and aggravated by the pandemic and the weakness of public policies and financial and economic crises, alerts us to the growing fragility of what remain the first bastions of access to healthcare for all (WHO, n.d.-a, n.d.-b).

### Invitations for further researches

The elements gathered here allow us to identify some initial limitations. The first concerns the lens of Western medicine, with the prevalence of the so-called modern hospital and the dual promotion of social hygiene and management. This approach needs to be contrasted with perspectives from other parts of the world, such as the Soviet hospital model(s) (Grant, 2015), or in the imperial circulations of the late nineteenth century. Numerous studies invite us to follow this path (Harrison, 2013; Nasim, 2023). The second limitation is that of gender. This could be overcome by drawing on developments in gender history, for example, which has documented the entry of women’s health into the international hospital agenda through women’s hospitals. We could also take advantage of the work done on women’s organizations within the League of Nations, particularly in relation to humanitarian issues (Piguet, Thébaud, 2020). The third limitation concerns the encounter between global hospital governance and environmental issues. This favors the emergence of transnational hospital issues: protocols for dealing with climate risks, such as heat waves, and civil society demands for compliance with energy and environmental standards. Indeed, hospitals are themselves sources of pollution. In 1987, the Goiânia (Brazil) accident showed that medical radiotherapy equipment can be a source of major nuclear accidents (IAEA, 1989). On a daily basis, hospitals face the challenge of standardizing the management of biological, toxic, and plastic waste, which must be historicized in the long term (Borowy, 2020). These few milestones in the history of global hospital governance invite us to consider it as a process of permanent adjustment between multiple stakeholders and a redefinition of their common interests. Like ports and factories, hospitals are destined to become one of the key objects in the history of contemporary globalization.
